# Dissociation and recovery in psychosis – an overview of the literature

**DOI:** 10.3389/fpsyt.2024.1327783

**Published:** 2024-04-05

**Authors:** Claudia Calciu, Rob Macpherson, Sui Yung Chen, Madalina Zlate, Rosemary C. King, Kerry J. Rees, Camelia Soponaru, Jackie Webb

**Affiliations:** ^1^ General Adult Psychiatry, Gloucestershire Health and Care NHS Foundation Trust, Gloucester, United Kingdom; ^2^ Psychology Faculty, School of Psychology and Educational Sciences, “Alexandru Ioan Cuza” University, Iasi, Romania; ^3^ General Adult Psychiatry, Birmingham and Solihull Mental Health NHS Foundation Trust, Birmingham, United Kingdom; ^4^ Later Life, Avon and Wiltshire Mental Health Partnership NHS Trust, North Somerset, United Kingdom; ^5^ Child and Adolescent Mental Health Service, Oxford Health NHS Foundation Trust, Oxford, United Kingdom; ^6^ Psychology Department, School of Natural and Social Sciences, University of Gloucestershire, Gloucester, United Kingdom

**Keywords:** dissociation, recovery, rehabilitation, psychosis, schizophrenia

## Abstract

**Background:**

The relationship between dissociation and recovery from psychosis is a new topic, which could attract the interest of the researchers in the field of dissociation due to its relevance to their daily clinical practice. This review brings together a diversity of international research and theoretical views on the phenomenology of dissociation, psychosis and recovery and provides a synthesis by narrative and tabulation of the existing knowledge related to these concepts.

**Aims:**

The objective was to make a synthesis by narrative and tabulation about what is known on the topic.

**Methods:**

The systematic search was conducted according to the PRISMA-statement in the databases Medline, PsycInfo, PubMed and Google Scholar. 2110 articles were selected according to the inclusion and exclusion criteria detailed in the methods, and 19 records were included in the review.

**Outcomes:**

None of the included publications put together, in the same conceptualisation or hypothesis, dissociation and the recovery from an episode of psychosis, therefore this matter remains unstudied at this time.

**Conclusion:**

The process of reviewing the existing scientific literature in the field of dissociation and recovery from psychosis has been very useful for charting the direction that future research will take.

## 1 Introduction

The NHS is going through a transformation process as it is moving to a more inclusive, flexible model of care, in which patients get properly joined-up care at the right time in the optimal care setting. The focus will be on prevention, inequalities reduction, and on responsiveness to all those who use and fund the health service. As part of the new model, there is a strong drive to invest in the transformation of dedicated community mental health rehabilitation functions (1). The concept of recovery is at the core of this transformation plan, associated with that of severe mental illness. This means that the model will need to be more differentiated in its support offer to individuals. Improving access to psychological therapies for those with severe mental health problems is a top priority on the transformation agenda (2).

The transformation process and the reshaping of the rehabilitation community services has to be founded on robust and up to date evidence-based data and research. This is the right time for new psychological interventions, more person tailored and more innovative, focusing on different concepts such as ‘trauma’, ‘dissociation’ and ‘recovery’, to be created.

For about a century, dissociative disorders and dissociative symptoms have been associated with trauma and traumatic experiences. There is a vast body of literature demonstrating the relationship between trauma and dissociative experiences, in longitudinal and prospective studies (3, 4). The meaning of dissociation has been a topic for scientific debate for a very long time and although there continue to be differences in opinion among clinicians and researchers, one aspect has been unanimously agreed upon; namely the fact that dissociation involves the loss of the ability of the mind to integrate some of its superior functions (4).

Whereas there is extensive scientific literature on the relationship between dissociation and psychosis (5–7) there is little if any on the topic of dissociative mechanisms and the process of recovery from psychotic episodes. Research in the field of recovery is difficult to undertake, but has included publications in the form of outcome studies, theoretical publications, meta-analyses, book chapters and conference presentations. The authors believed that the process of reviewing the existing scientific literature in the field of dissociation and recovery from psychosis may be useful to understand the current state of knowledge and for charting the direction of future research.

## 2 Concepts

### 2.1 Dissociation

Dissociation is “*the fragmentation of the usual continuity of subjective experience*”;^8^ “*disruption of and/or discontinuity in the normal integration of conscience, memory, identity, emotion, perception, body representation, motor control and behaviour*” (8) “*partial or total loss of the normal integration between the memory of the past, identity awareness and body movements control*” (9). This idea derived from the concept of “disintegration” of the integrative function, introduced by Pierre Janet (1859-1947). This would result in a process of fragmentation at different mental levels, from consciousness to the personality unity itself (10). As opposed to Freud’s theory which defined dissociation as a defensive mechanism, Janet associated it with the loss of the connexion between normally integrated and overlapped mental functions, due to a “structural collapse” caused by traumatic experiences (10).

### 2.2 Psychosis

“The term “psychosis” lies at the heart of modern psychiatry” (11). DSM (8) and ICD-10 (9) describe specific diagnostic criteria for different psychotic conditions. Sadock and colleagues define the concept of psychosis as a group of mental illnesses where the loss of reality testing and the boundaries of the self are the main characteristics (12). Schizophrenia is often referred to in the specialist literature as representative for the psychosis group. It is a serious mental illness where the misinterpretation of stimuli from the external environment influences the information processing. As a result, a series of abnormal phenomena will occur in the form of positive symptoms (delusions and hallucinations), negative symptoms (apathy, anhedonia, dull affect, and loss of social cohesion), and cognitive ones.

Although distinctive symptoms for schizophrenia and dissociative disorders are listed by both ICD-10 and DSM-5, studies have shown an overlap between psychotic symptoms (for example, auditory hallucinations) and dissociation manifestations (13, 14). The causal relationship between dissociation and psychosis remains unexplored (3). Cernis and colleagues are of the view that this may be due to a possible lack of clarity about the role of dissociation in mental health (15).

### 2.3 Recovery

There are different points view of recovery but in this paper, the definition we used is the one coined by Anthony (16), who defined recovery as “*a deeply personal, unique process of changing one’s attitudes, values, feelings, goals, skills and roles. It is a way of living a satisfying, hopeful, and contributing life even with limitations caused by the illness. Recovery involves the development of new meaning and purpose in one’s life as one grows beyond the catastrophic effects of mental illness*”. For this review, we aimed to identify articles that defined personal recovery.

## 3 Aim of the literature review

The aim of this review is to conduct an evaluation of the literature on recovery from psychosis and dissociation, using broader inclusion criteria thereby providing a synthesis by narrative and tabulation about what is known on the topic.

## 4 Methods

### 4.1 Search strategy

We limited the search to papers in the English language as we did not have access to volunteer or paid interpreters. We restricted the search to papers referring to population of any age within the interval 18-65. Medline, PsycInfo, PubMed databases were systematically searched using strings for dissociation, psychosis and recovery concepts: (dissociat* OR compartmentali* OR detach* OR absorption OR depersonalisation OR derealisation OR amnesia* OR “coping mechanism*” OR fragment*) AND (psychosis OR psychotic OR hallucinat* OR delusion* OR “positive symptom*” OR “negative symptom*” OR schizophreni* OR “thought disorder*” OR schizoaffective) AND (recover* OR outcome* OR recuperat* OR rehabilitat* OR improve* OR hope*). Google Scholar database was also searched using the following search strategy: Articles with any of these words in the article (Dissociate/compartmentali/psychosis/schizophrenia/psychot/hallucinate/recover/rehabilitat/outcome) or with these words in the title (psychosis/dissociation/therapeutic). 14 studies were identified using this method.

Beside the database search, a hand-search of references and citations from eligible articles was also performed in order to identify additional studies. Five studies were included using this method.

**Figure 1:**
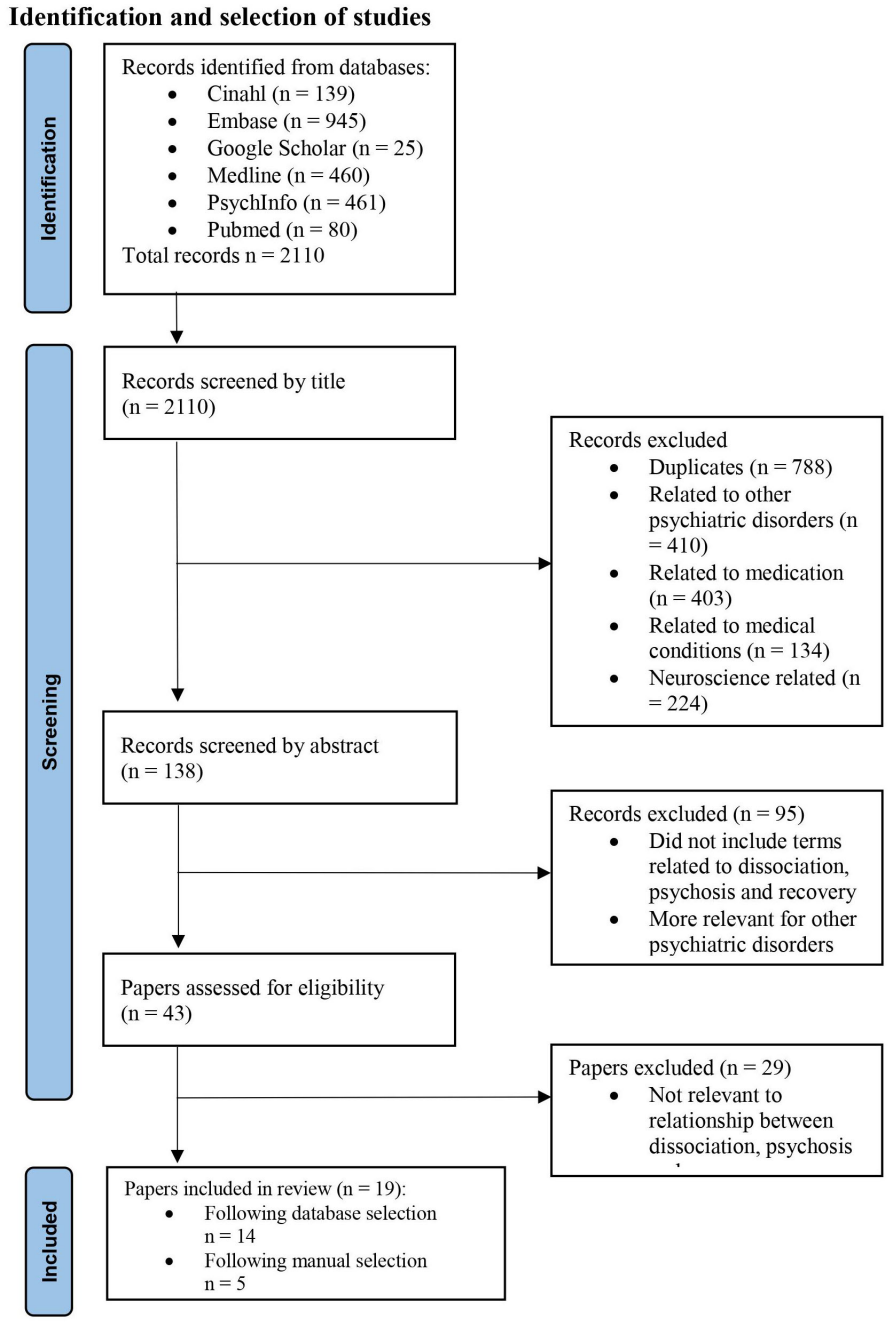
Study selection.

Articles were assessed for eligibility based on screening of titles, abstracts and full texts and only retained for review with consensus agreement from four reviewers. The search and screening procedure are presented in **Figure 1**.

### 4.2 Inclusion and exclusion criteria

Inclusion criteria:

1. Publications on dissociation and psychosis and recovery, including terms (utilised in the search strategy) such as:

• Dissociation – compartmentalisation, detachment, absorption, depersonalisation, derealisation, amnesia, copying mechanism, fragmentation.
• Psychosis - hallucinations, delusions, positive symptoms, negative symptoms, schizophrenia, thought disorder, schizoaffective, psychotic, delusional.
• Recovery – outcome, recuperation, rehabilitation, improvement, hopefulness.

2. Articles published in English;

3. No date or age limits;

4. Studies on clinical and/or non-clinical population.

Exclusion criteria:

1. Articles about dissociation and psychiatric conditions other than psychosis;
2. Articles about dissociation and medical conditions;
3. Studies from the neuroscience domain.

### 4.3 Identification and selection of studies

Following the selection process, 19 articles were included in this review. They are described in **Table 1**.

**Table 1:** Description of articles.

Nr	Article - full citation	Country	Study design	Aim	Population	N	Age	Recruitment method
1	Longden, E.; Branitsky, A.; Moskowitz, A.; Berry, K.; Bucci, S.;Varese, F. The Relationship between dissociation and symptoms of psychosis: A Meta-analysis, Schizophrenia Bulletin.2020 doi:10.1093/schbul/sbaa037		Meta-analysis	To quantify the magnitude of association between dissociative experiences and all symptoms in psychosis.	Clinical and non-clinical population	20436	Mean age 27.07	
2	Farrelly, S.; Peters, E.; Azis, M.; David, A.; Hunter, E.C. A brief CBT intervention for depersonalisation/derealisation in psychosis: study protocol for a feasibility randomised controlled trial, Pilot and Feasibility Studies. 2016 2:47.	UK	Single-blinded RCT	To determine the feasibility and acceptability of a brief CBT intervention for clinically significant depersonalisation in people with psychotic symptoms	clinical population	30	18-70	Secondary mental health trust – community mental health teams, psychological therapies services and research registers.
3	Hwu, H.G.; Chen, C.C.; Tsuang, M.T.; Tseng, W.S. Derealization syndrome and the outcome of schizophrenia: A report from the international pilot study of schizophrenia, British Journal of Psychiatry. 1981, 139,313-318.		Two-year follow-up from the International Pilot Study of Schizophrenia (IPPS, WHO, 1973)- transcultural psychiatric investigation	Prognostic implication of the clinical manifestations of derealisation at initial evaluation in relation to outcome at two-year-follow-up from the Pilot Study of Schizophrenia (IPPS) (WHO, 1973)	clinical population	133 in follow up study (137 original study)	15 +	
4	Perona-Garcelan, S.; Cuevas-Yust, C.; Garcıa-Montes, J.M.; Perez-Alvarez, M.; Ductor-Recuerda, M.; Salas-Azcona, R.; Gomez-Gomez, M.T.; Rodrıguez-Martın, M.&B. Relationship Between Self-Focused Attention and Dissociation in Patients With and Without Auditory Hallucinations, The Journal of Nervous and Mental Disease. 2008;196: 190–197.	Spain	Cross-sectional	To study the relationship between self-focussed attention and dissociative experiences.	clinical population and non-clinical control group	68	20 - 62 (Mean age 38.65, SD 9.04)	Patients with auditory verbal hallucinations, with ICD 10 diagnosis. Attending the mental health units of the Virgen del Rocío Hospital (Seville, Spain), the Sierrallana Hospital (Santander, Spain) and the San Carlos Clinical Hospital (Madrid, Spain). Control grup - staff and trainees from the Virgen del Rocío Hospital (Seville, Spain).
5	Perona‐Garcelán, S.; García‐Montes, J. M.; Ductor‐Recuerda, M. J.; Vallina‐Fernández, O.; Cuevas‐Yust, C.; Pérez‐Álvarez, M.; Salas-Azcona, R.; Gómez‐Gómez, M. T. Relationship of metacognition, absorption, and depersonalisation in patients with auditory hallucinations, British Journal of Clinical Psychology. 2012, 51, 100-118.	Spain	Cross-sectional	To study the relationship of metacognition, absorption, and depersonalisation in hallucinating patients.	clinical population and non-clinical control group	124	16-65 (Mean age 37.9; SD 9.92)	“Patients diagnosed with psychosis, selected from those receiving attention at the Virgen del Roc´ıo Hospital in Seville (southern Spain) or the Sierrallana Hospital in Torrelavega (northern Spain), and treated with neuroleptic medication. The patients in the clinical control group were receiving attention at the outpatient health services or from private psychologists.
6	Wright, A.; Fowlerb, D.; Greenwood, K. Influences on functional outcome and subjective recovery in individuals with and without First Episode Psychosis: A metacognitive model, Psychiatry Research. 2020, 284, 112643.	UK	Cross-sectional	Association of metacognition and subjective recovery in first episode of psychosis	clinical population and non-clinical control group	135	Clinical group18-43 (mean age 26.24, SD 5.66) Control (mean age 26.3, SD 6.6)	Individuals with psychosis were recruited through a convenience sample from Early Intervention in Psychosis services in Sussex Partnership NHS Foundation Trust, with a minority of these from a previous first episode psychosis (FEP) sample. Healthy control participants were recruited through advertisement within the local community, for example, in libraries and cafes, and online, for example, through social media and Gumtree.
7	Rosen, C.; Jones, N.; Chase, K.A.; Melbourne, J.K.; Grossman, L.S.; Sharma, R.P. Immersion in altered experience: An investigation of the relationship between absorption and psychopathology, Consciousness and Cognition. 2017, 49, 215–226.	USA	Cross-sectional	To explore the phenomenological construct of absorption and psychotic experiences in clinical and non-clinical participants.	clinical population and non-clinical control group.	115 (76 African American, 10 Asian, 19 Caucasian, 10 Hispanic)	21-60	Population randomly selected from a large urban university medical centre but included referrals from community treatment facilities. Non-clinical participants were recruited from neighbouring communities.
8	Úbeda-Gómeza, J.; León-Palaciosa, M.G.; Escudero-Péreza, S.; Barros- Albarrána, M.D.; López-Jiménezb, A.M.; Perona-Garcelána, S. Relationship between self-focussed attention, mindfulness and distress in individuals with auditory verbal hallucinations, Cognitive Neuropsychiatry. 2015, 20: 6, 482–488.	Spain	Cross-sectional	To investigate the relationships among self-focussed attention, mindfulness and distress caused by the voices in psychiatric patients.	Clinical population	51	18-65 (mean 38, DS 10.24)	The participants were inpatients in the mental health units of the Virgen del Rocío Hospital (Seville, Spain), the Sierrallana Hospital (Santander, Spain) and the San Carlos Clinical Hospital (Madrid, Spain).
9	“Humpston, C.S.; Walsh, E.; Oakley, D.A.; Mehta, M.A.; Bell, V.; Deeley, Q. The relationship between different types of dissociation and psychosis-like experiences in a non-clinical sample, Consciousness and Cognition. 2016, 4, 83–92. “	UK	Cross-sectional	To investigate whether detachment, compartmentalisation or absorption were most strongly associated with psychosis-like experiences in the general population.	Non-clinical population	215	18-67 (Mean age 27.16; SD9.28)	General population sample recruited through adverts placed on the gumtree.com website in London and from an email circular that was distributed to all staff and students at three central London universities.
10	Lynch, S.; Holttum, S.; Huet, V, The experience of art therapy for individuals following a first diagnosis of a psychotic disorder: a grounded theory study, International Journal of Art Therapy. 2018, 24:1, 1-11.	UK	Qualitative research	To explore how service users experienced art therapy following their first diagnosis of a psychotic disorder, and the processes through which art therapy might be helpful for such individuals.	Clinical population	8	24-52 (Mean age 34.75)	Participants were recruited through art therapists in 4 NHS mental health trusts in southern UK. Interviews took place either face-to-face or by telephone and were audio-recorded.
11	Bacon, T. & Kennedy, A. Clinical perspectives on the relationship between psychosis and dissociation: utility of structural dissociation and implications for practice, Psychosis. 2014, 7:1, 81-91.	Patients from England, Scotland, Sweden, Germany, USA	Qualitative research	To present a qualitative research project that explored practice-based perspectives on the relationship between psychosis and dissociation. To conceptualise the model of Structural Dissociation of the Personality		10		8 - The International Society for the Psychological and Social Treatments of Psychosis 2- The European Society for the Study of Trauma and Dissociation
12	Ross, C.A.; Keyes, B.B., Clinical features of dissociative schizophrenia in China, Psychosis. 2009,1:1, 51-60.	China	Mixed - Qualitative study and case reports	To describe some clinical examples of dissociative schizophrenia from China. To describe the dissociative subtype, and to demonstrate that it occurs outside North America, where most of the research supporting the existence of the subtype has been conducted.	Clinical population	50	19 - 70 (Mean age 43.3; SD 12.4)	All were inpatients at Shanghai Mental Health Center and had clinical diagnoses of schizophrenia made by their attending psychiatrists using Chinese diagnostic criteria.
13	Lysaker, P.H.; Minor, K.S.; Lysaker, J.T.; Hasson-Ohayon, I.; Bonfils, K.; Hochheiser, J.; Vohs, J.L.Metacognitive function and fragmentation in schizophrenia: Relationship to cognition, self-experience and developing treatments, Schizophrenia Research: Cognition. 2020,19 100142.		Literature overview/ summary of research on quantifying metacognition	To review research seeking to measure some of the aspects of fragmentation related to the experience of the self and others				
14	Lysaker, P.H.; Hamm, J.A.; Vohs, J.; Kukla, M.; Pattison, M.L.; Leonhardt, B.L.; Lysaker, J.T. Understanding the course of self-disorders and aterations in self-experience in schizophrenia: implications from research on metacognition, Current Psychiatry Reviews. 2018, 14, 160-170.		Literature review/theoretical models	To review research on the integrated model of metacognition in schizophrenia and explore five descriptions of alterations in subjective experience, which are sometimes called self-disorders.				
15	Kumar, D.; Venkatasubramanian, G. Metacognition and mindfulness integrated therapy reduces severity of hallucination in a patient not taking antipsychotic medication, Journal of Cognitive Psychotherapy. 2018, 32: 3,192-202.	India	Case report	Efficacy of metacognition & mindfulness integrated therapy in reduction of hallucination of patients not taking antipsychotic medication.	Clinical population	1	55	
16	Perivoliotis, D.; Grant, P.M.; Beck, A.T. Advances in Cognitive therapy for schizophrenia: Empowerment and recovery in the absence of insight, Clinical Case Studies. 2009, 8(6) 424-437.	USA	Case report	To describe a cognitive therapy approach innovated to circumvent limited insight in a patient with severe paranoia and auditory hallucinations	Clinical population	1	24	Not specified, although parents encouraged her to enrol
17	Pec, O.; Lysaker, P.H.; Probstova, V.; Leonhardt, B.L.; Hamm, J.A.; Bob, B. The psychotherapeutic treatment of schizophrenia: Psychoanalytical explorations of the metacognitive movement, Journal of Contemporary Psychotherapy. 2020, 50, 205–212.		Theoretical (psychoanalytical conceptualisation)	To explore how psychoanalytic theory can explain how the effects of MERIT upon metacognition and self-experience in schizophrenia may reflect its effects on repairing the collapse of the boundary/connection between self and the world, mental fragmentation and the lack of symbolisation.				
18	Bob, P. & Mashour, G.A. Schizophrenia, dissociation and consciousness, Consciousness and Cognition. 2011, 20, 1042-1049.		Theoretical	A review of findings on dissociation, conscious disintegration and schizophrenia				
19	Ross, C. A. (2006). Dissociation and psychosis: The need for integration of theory and practice. In J. O. Johannessen, B. V. Martindale, & J. Cullberg (Eds.), Evolving psychosis (pp. 238–254). Routledge/Taylor & Francis Group.		Theoretical- Book chapter	To point out logical and scientific errors in the dominant conceptual system of psychosis and dissociation.				

### 4.4 Quality assessment

Studies were reviewed by four authors individually. Disagreements related to the eligibility of the studies were resolved by finding additional information and through discussions between the authors. Due to the diverse designs of the studies and articles included, a narrative synthesis approach was employed in order to obtain a summary of the information and data encompassed by the selected literature.

### 4.5 Data extraction

Data extraction was conducted by three authors and systematically checked for accuracy by the main author. Information extracted from the primary studies was recorded on a standardised form including general characteristics (authors, publication title, country, publication year), design, sample characteristics (age, clinical/non-clinical, recruitment method), measures used to assess dissociation, psychosis and other dimensions, who applied the instruments and the limitations of the studies.

## 5 Results

### 5.1 Study design

The design and the type of included studies and articles were diverse, including: a randomised controlled trial (17), a meta-analysis (7), a longitudinal study (18), seven cross-sectional studies (6, 19–24), two qualitative studies (5, 25), one mixed methods design (26), two literature reviews (27, 28), a case report (29), two articles summarising theoretical views (30, 31), and a book chapter (32).

The included studies and articles are described in **Table 1**, which has inevitably some incomplete sections as the information that would have populated the respective sections, was not reported in the publications included.

### 5.2 Definitions used in the included publications

There are different conceptualisations of the notions on which we focussed our review. We did not analyse them as they were not always reported in the included articles, therefore we thought appropriate to presented them as used in the studies that reported them.

We looked at how the papers defined the keywords included in the database search (dissociation, psychosis and recovery) and the associated terms (compartmentalisation, detachment, absorption, depersonalisation, derealisation, amnesia, copying mechanism, fragmentation, hallucinations, delusions, positive symptoms, negative symptoms, schizophrenia, thought disorder, schizoaffective, psychotic, delusional, outcome, recuperation, rehabilitation, improvement, hopefulness). Most commonly defined terms were schizophrenia, psychosis, dissociation, compartmentalisation, and absorption.

The term psychosis as a broader concept is used in three papers (5, 17, 25). Most of them use the notion of schizophrenia as representative for psychosis (18, 22, 24, 27, 28, 31, 32). None of the papers include definitions for terms related to recovery or rehabilitation. Five publications do not include any definitions of the concepts relevant to this paper (20, 21, 23, 30, 32).

The concept of dissociation was defined either as a general concept or by referring to specific dissociative mechanisms. Longden and colleagues refer to the DSM-5 definition of dissociation as ‘*‘a disruption of and/or discontinuity in the normal integration of consciousness, memory, identity, emotion, perception, body representation, motor control and behaviour’’* (7, 8). Humpston and colleagues look at which type of dissociation is most associated with psychosis-like experiences (6). They refer to the notions of “*compartmentalisation-type dissociation which stems from the work of Pierre Janet [ … ] who originated the modern concept of dissociation as the compartmentalisation of normally integrated mental functions leading to the loss of conscious control or awareness of specific mental, physical or sensory processes*” (33) and absorption “which relates to the ability to become immersed in thoughts and experiences’’ (6, 34).

Farelly and colleagues define psychosis as “a general term covering a range of psychiatric diagnoses such as schizophrenia, schizoaffective disorder and delusional disorder” (17) and dissociation “*as a disruption of and/or discontinuity in the normal integration of consciousness, memory, identity, emotion, perception, body representation, motor control and behaviour”* (17), as per DSM-5 (8). Further, they offer definitions for detachment: “(it*) concerns a person’s sense of separation from experience, including from their sense of self [ … ] or from the external world*’’ and for compartmentalisation: “*a disruption in normally integrated functions that is not accessible to conscious control and includes dissociation*’’ *(* 17).

Rosen and colleagues talk about the “*ipseity of schizophrenia* (the term refers to the nature of self in schizophrenia), *involving two core components of disturbed basic sense of self: hyperreflexivity and diminished self-affection* (22). Hyperreflexivity refers to the process by which events, sensations and cognitions that would normally be experienced as tacit [ … ] become explicit’’ and “diminished self-affection described the loss of attenuation of a normal sense of the self-existing as the subject [ … ] of consciousness” (22). They also define the concept of absorption, which “describes a state of immersion in (or capture by) mental imagery or perceptual stimuli and correlates vivid imagination or fantasy’’ (22).

Bacon and Kennedy differentiate between “psychosis-as-PTSD’’ and “psychosis-as-dissociation’’ (5). Focusing on the term relevant to this paper (dissociation), they further explain that in the case of ‘‘psychosis-as-dissociation’’ is ‘‘where psychotic symptoms represent interplay between deeply fragmented and incohesive ego-states, and the deterioration of the ego” (5).

Bob and Mashour refer to Eugen Bleuler’s definitions of schizophrenia: “in 1911 Eugen Bleuler introduced the term schizophrenia as a description of this mental illness [ … ], which replaced Kraepelin’s term dementia praecox’’ (31). For the term dissociation they mention several definitions, from Pierre Janet and Bleuler’s definition: “Janet used the term dissociation to denote a splitting of the psyche and analogously Bleuler [ … ] used the term dissociation as a synonym for splitting’’ to a more recent definition: “the recent definition of dissociation as a special form of consciousness in which events that would ordinarily be connected are divided from one another, leading to a disturbance or alteration in the normally integrative functions of identity, memory, consciousness” (31).

Lysaker and colleagues discuss the concept of schizophrenia from different perspectives: as defined by Kraepelin, Bleuler, Rosenbaum (“disconnection of images, affects and ideas and causes a breach in the unity of the self and threatens the many symbolic link characterising its integrating capacities and its reality testing”), from a psychoanalytic point of view, from an existential perspective with Laing’s focus on subjective experience of schizophrenia (“fundamental kind of alienation or a rent in his relation with his world [ … ] and a disruption of his relation with himself”), the phenomenological and ipseity model of self-experience in schizophrenia, the rehabilitation and recovery based models of disturbance in self-experience in schizophrenia, and the dialogical models of schizophrenia (27, 28).

Two articles refer to the definitions and diagnostic criteria from DSM-IV-TR and the American Psychiatric Association for a dissociative subtype of schizophrenia (32, 35). Perivoliotis and colleagues define schizophrenia as a “chronic disorder associated with significant disability and poor quality of life” (24). Hwu and colleagues refer to Eugen Bleuler’s definition of schizophrenia “as a constellation of fundamental symptoms” (18). Lynch and colleagues mention that “there are different ways of conceptualising ‘psychosis’ or ‘psychotic experiences’ (25) which include hearing voices and having unusual beliefs’’ (36).

Perona-Garcelan and colleagues use terms such as dissociation, hallucinations, schizophrenia, absorption, depersonalisation, however they do not define them (20).

Detachment and absorption are two dissociative phenomena explored by some of the studies included in this review. Detachment refers to a mental process also sometimes termed depersonalisation/derealisation. These phenomena encompass the experience of detachment from self and environment where the self and the environment are experienced as unfamiliar or altered (37). DSM-5 describes depersonalisation/derealisation as a dissociative disorder per se and also as a symptom characterising other psychiatric conditions (8).

Other authors describe a more “normal” aspect of dissociation. Buttler introduces the idea of “normal” dissociation and describes absorption as representative for this type of dissociation (38). Absorption is very often referred to as “normal” or non-pathological dissociation (39). It represents the involuntary tendency to attention narrowing to the extension of ignoring the environment and implies a temporary suspension of the reflective consciousness (38).

### 5.3 Participants

14 studies of the 19 publications selected for this review, included population samples of different sizes (see **Table 1**), the total number of participants being 21.377. The study by Logden was substantially the largest (7). They were recruited from the adult population aged between 18 and 70, with two studies recruiting younger participants (18, 20). The majority of the studies recruited participants from the clinical population. Seven studies were developed on clinical popuations only (17, 18, 23–26, 29). One cross-sectional study included only a non-clinical population (6). Five studies selected their control groups from a non-clinical population (7, 19–22). One study does not report which population the participants were recruited from (5).

### 5.4 Assessment tools

The 19 studies included in our review encompass a wide variety of tools. Out of them, one appears in a third of the studies, namely the Positive and Negative Syndrome Scale (PANSS) (40). A total of 33 measuring instruments including clinical interviews were used (see **Table 2**), which we grouped into the following categories:

**Table 2:** Classification of instruments.

Nr	Article – author/year	Instruments			Who applied
		Psychosis/other psychopathology	Dissociation	Other	
1	Longden et al. (2020)”	PANSS (Kay, Fiszbein & Opler, 1988); not all mentioned	DES-II (Bernstein & Putnam, 1986); not all mentioned	not mentioned	
2	Farrelly et al. (2016)	PSYRATS (Haddock et al., 1999)	CDS (Sierra & Berrios, 2000)	BDI (Beck, Steer & Brown, 1996); BAI (Beck & Steer, 1990), PDS (Foa et al., 1997), SCID-D (Steinberg, 1994)	Researcher
3	Hwu et al. (1981)				
4	Perona-Garcelan et al. (2008)	PANSS (Kay, Fiszbein & Opler, 1988)	DES-II (Bernstein & Putnam, 1986)	SCS-R (Scheier & Carver, 1985)	Clinical psychologist
5	Perona-Garcelan et al. (2012)	PANSS (Kay, Fiszbein & Opler, 1988)	TAS (Tellegen & Atkinson, 1974); CDS (Sierra & Berrios, 2000)	MCQ-30 (Wells & Cartwright- Hatton, 2004)	Self-report (TAS) Self-administered (CDS) Administered by the clinical psychologist who was responsible for the patient’s care (PANSS)
6	Wright et al. (2020)	PANSS (Kay, Fiszbein & Opler, 1987)		Computer based visual and auditory detection tasks; The cognitive insight scale (Beck et al., 2004), The Metacognitive Assessment Interview (Semerari et al., 2012), Time Use Survey (Short, 2006), The UCSD Performance-Based Skills Assessment (Patterson et al., 2001), The Questionnaire of Process of Recovery (Neil et al., 2009), The Wechsler Abbreviated Scale of Intelligence (Wechsler 1999)	
7	Rosen et al. (2017)	SCID (First et al., 2002) PANSS ((Kay, Fiszbein & Opler, 1988)	TAS (Tellegen & Atkinson, 1974);		
8	Úbeda-Gómez et al. (2015)	PSYRATS (Haddock et al., 1999)	SAS (Mckenzie & Hoyle, 2008)	MAAS (Brown & Ryan, 2003)	
9	Humpston et al. (2016)	PDI (Peters et al., 2004); CAPS (Bell, Halligan &Ellis, 2006)	DES (Bernstein &Putnam, 1986); TAS (Tellegen & Atkinson, 1974)	HGSHS: A (Shor &Orne, 1962)	HGSHS: A was administered at group level, then each participant was given a questionnaire pack containing the HGSHS: A, the PDI-21, the CAPS, the TAS and the DES
10	Lynch et al. (2018)			Semi-structured interview developed in discussion with the other two authors, with open and non-leading questions, and adapted as the research progressed, in line with grounded theory methodology.	First author (Clinical psychologist)
11	Bacon & Kenedy (2014)			Interpretative Phenomenological Analysis (IPA; Smith, Flowers, & Larkin, 2009) of semi-structured telephone interviews - detailed scrutiny of interview transcripts, development of conceptual themes, and repeating this with each set of interview data before superordinate and subordinate themes accommodating all experiences were produced.	
12	Ross & Keyes (2009)			Clinical interview	The authors (The histories were taken with a psychiatry resident acting as translator)
13	Lysaker et al. (2020)			MAS-A (Lysaker et al., 2005)	
14	Lysaker et al. (2018)				
15	Kumar et al. (2018)	PSYRATS (Haddock et al., 1999)			clinician
16	Perivolioitis et al. (2009)	PSYRATS (Haddock et al., 1999), SANS (Andreasen, 1984), BAVQ-R (Chadwick et al., 2000)		BDI -II (Beck, Steer & Brown, 1996); BAI (Beck & Steer, 1990); Strauss-Carpenter Levels of Function Scale (Strauss & Carpenter, 1974); QoL Inventory (Frisch, 1994); BCIS (Beck et al., 2004)	Assessors blind to treatment condition; Self-report; Administered by therapist during therapy
17	Pec et al. (2020)				
18	Bob & Mashour (2011)				
19	Ross (2206)				

• Scales for the measurement of psychosis (see **Table 3**).

**Table 3:** Instruments used to measure psychosis.

Nr	Instrument, Author	Items/Time/Formats	Domains assessed	Reliability	Validity
1	PANNS *(Kay, Fiszbein & Opler, 1988)* 5 studies: Perona-Garcelan et al., 2008, 2012; Wright et al., 2020; Rosen et al., 2017; Longden et al., 2020	30 items Positive scale items (7) Negative scale items (7) General psychopathology scale items (16) observations from interview/verbal report/information from care givers Time: 1 hour Response: PANNS rating anchors 1-7 (absent to extreme)	*Psychotic symptoms:* *positive symptoms (*delusions, grandiosity suspiciousness/persecution, unusual thought content) *negative symptoms* (blunted affect, emotional withdrawal, poor rapport, passive/apathetic social withdrawal, lack of spontaneity and flow of conversation, active social avoidance	Reported: *Rosen et al.,2017* Internal consistency α = 0.77 – 0.89 Test-retest reliability r=0.80 Inter-rater reliability – Kappa = 0.85 Not reported: *Perona-Garcelan et al., 2008, 2012;* *Wright et al., 2020;* *Longden et al., 2020*	Not reported by any of the 5 studies
2	PSYRATS (*Haddock et al., 1999*) 4 studies: Kumar et al., 2018; Ubeda-Gomez et al., 2015; Perivoliotis et al., 2009; Farrelly et al., 2016	Interviewer -scored 2 subscales: hallucinations subscale (11 items); delusions subscale (6 items) Response: 5-point Likert-type scale (0 ‘not present’ -4’present continuously’)	Measures various dimensions (presence, typology, beliefs/conviction, distress and disruption associated) of delusions and auditory hallucinations	Reported: *Ubeda-Gomez et al., 2015* Inter-rater reliability 0.80 Not reported: Kumar et al., 2018; Perivoliotis et al., 2009; Farrelly et al., 2016	Not reported by any of the 4 studies
3	SCID (*First et al., 2002*) 1 study: Rosen et al., 2017	Structured clinical interview for the DSM Response: 3 point scale (1- absent; 2- subthreshold; 3 – threshold or true present)	Psychopathological assessments of types of hallucinations and forms of delusions	Inter-rater reliability Kappa 0.83	Not reported
4	SANS (*Andreasen, 1984)* 1 study: Perivoliotis et al., 2009	25 items Interviewer-scored scale	Negative symptoms of schizophrenia	Not reported	Not reported
5	BAVQ-R (*Chadwick et al., 2000)* 1 study: Perivoliotis et al., 2009	Not described	Auditory hallucinations	Not reported	Not reported
6	PDI (Peters et al., 2004) 1 study: Humpston et al., 2016	21 item- scale	Delusional ideation	Cronbach’s alpha 0.77	Not reported
7	CAPS (bell, Halligan & Ellis, 2006) 1 study: Humpston et al., 2016	32 items- scale Subscales: Distress, intrusiveness, frequency	Anomalous perceptual experiences psychosis-like	Cronbach’s alpha 0.87	Good construct validity

• Scales for the measurement of dissociative experiences (see **Table 4**).

**Table 4:** Instruments measuring dissociation.

Nr	Instrument, Author	Items/Time/format	Domains assessed	Reliability	Validity
1	DES-II *(Bernstein & Putnam, 1986)* 3 studies: Perona-Garcelan et al., 2008; Humpston et al., 2016; Longden et al., 2020	28 questions Self-test Response: 11-point percentage scale (0% “never” to 100% “always”) 3 subscales: amnesia, absorption and detachment	Dissociation – frequency of experiences: Amnesia, Absorption/imaginative involvement, Depersonalisation/derealisation	Reported: *Perona-Garcelan et al., 2008* Internal consistency α = 0.91 *Humpston et al., 2016* Internal consistency α = 0.95 Not reported: *Longden et al., 2020*	Reported: *Humpston et al., 2016* “good construct validity” Not reported: *Perona-Garcelan et al., 2008* *Longden et al., 2020*
2	TAS *(Tellegen & Atkinson, 1974)* 3 studies: Perona-Garcelan et al., 2012; Rosen et al., 2017; Humpston et al., 2016	34-item 5 subscales: synaesthesia, altered state of consciousness, aesthetic involvement, imaginative involvement and extrasensory perception Self-report Response: 5-point Likert scale (0 ‘never’ to 4 ‘always’)	Levels of mental involvement with the object of experience	Reported: *Perona-Garcelan et al., 2012* Internal consistency α = 0.93 *Rosen et al., 2017* Internal reliability r=0.88 Test-retest reliability r=0.91 *Humpston et al., 2016* Internal consistency α =0.93	Not reported by any of the 3 studies
3	CDS *(Sierra & Berrios, 2000)* 2 studies: Perona-Garcelan et al., 2012; Farrelly et al. (2016)	29 items Self report Response: 2 Likert-type scales: 1.frequency of the experience, from 0 (never) to 4 (always) 2.Duration of the depersonalisation experience from 0 (a few seconds) to 6 (over a week)	descriptive evaluation of depersonalisation experiences	Reported: *Perona-Garcelan et al., 2012* Cronbach’s alpha of.947 for the total scale score,.937 for the frequency scale and.943 for the duration scale. Not reported: *Farrelly et al. (2016)*	Not reported by either of the 2 studies
4	SAS (*Mckenzie & Hoyle, 2008)* 1 study: Ubeda-Gomez et al., 2015	17 items scale 2 subscales: Private self-absorption (8 items); Public self-absorption (9 items) Response: 5-point Likert scale (0 ‘never’ to 4 ‘always’)	The level of self-absorption	Internal consistency α =0.85 (private) and 0.91 (public)	Not reported

• Other scales (see **Table 5**).

**Table 5:** Other instruments.

Nr	Instrument, Author	Items/Time/format	Domains assessed	Reliability	Validity
1	Semi-structured interview 1 study: Lynch et al., 2018	Face-to-face or telephone & audio recorded Time: 20 – 51 minutes Response: Grounded theory	Experience of art therapy following first diagnosis of psychiatric disorder Processes through which art therapy might help *Psychosis*	Qualitative	Qualitative
2	Clinical interview 1 study: Ross & Keyes, 2009	Clinical interviews	*Dissociation* *Dissociation subtype*	Qualitative	Qualitative
3	SCS-R *(Scheier & Carver, 1985)* 1 study: Perona-Garcelan et al., 2008	22 items 3 subscales: Self-focussed attention items (9) Public self-consciousness items (7) Social anxiety items (6) Response: 4-choice answer (completely agree to completely disagree)	Self-consciousness (self-focussed attention) as a trait or disposition	Internal consistency α= 0.92, 0.75, and 0.81 for each of the subscales, respectively	Not reported
4	MCQ-30 *(Wells & Cartwright-Hatton, 2004)* 1 study: Perona-Garcelan et al., 2012	30 items questionnaire Five factors: ‘Loss of cognitive confidence’ ‘Positive beliefs about worry’ ‘Cognitive self-consciousness’ ‘Negative beliefs about uncontrollability and danger’ ‘Need to control thoughts’ Response: a scale of 1 (do not agree) to 4 (completely agree)	A range of metacognitive domains which are important in conceptualising psychopathological processes	Reliability for each of the 5 factors: ‘Cognitive confidence’ α = 0.85 ‘Positive beliefs’ α = 0.84 ‘Cognitive self-consciousness’ α = 0.75 ‘Uncontrollability and danger’ α = 0.79 ‘Need to control thoughts’ α = 0.78	“The construct and convergent validity are supported by empirical studies”
5	MAS-A (*Lysaker et al., 2005)* 1 study: Lysaker et al., 2020	A rating scale -not described in the paper	Metacognition as it is apparent within personal narratives *Metacognitive acts* *Metacognitive knowledge*	Not reported	Not reported
6	Computer based visual and auditory tasks 1 study: Wright et al., 2020	Task: to make 2 forced-choice binary judgements of 1.a) whether a stimulus was present or not within a noisy picture or presentation of white noise; 2.b) whether confidence in this decision was high or low; 2) to discriminate between correct and incorrect judgements	Metacognition efficiency Metacognitive sensitivity Metacognitive experience		
7	CIS (*Beck et al., 2004*) 1 study: Wright et al., 2020	9 items Self-reflective subscale of the cognitive insight scale	Metacognitive monitoring	Internal consistency α =0.68; test-retest reliability r= 0.90	Convergent validity r = -0.67
8	MAI (*Semerari et al., 2012*) 1 study: Wright et al., 2020	Requires the participant to reflect on a recent difficult interpersonal experience and answer a series of questions	Metacognitive ability	Internal consistency α =0.90; reliability r= 0.62 to 0.90	Good factorial validity
9	TUS (*Short, 2006*) 1 study: Wright et al., 2020	Structured interview Questions re the number of hours spent engaged in specific structured activities for the preceding month	Functional outcome	Inter-rater reliability 0.99	Good validity as TUS is comparable to studies using functioning measures
10	The UCSD Performance-based Skills Assessment (*Patterson et al., 2001*) 1 study: Wright et al., 2020	Total score for real-life performance skill based on role-play tasks. 0-20 scale 5 Sections: *Finance* *Communication* *Planning* *Transport* *Household*	Functional capacity	Internal consistency α =0.88; test-retest reliability r= 0.91	Validity r= 0.86
11	QPR (Neil et al., 2009) 1 study: Wright et al., 2020	22 items Self -reported questionnaire 2 subscales: Interpersonal subscale; Intrapersonal subscale Response: a scale from 0 (strongly disagree) to 4 (strongly agree)	Individual’s subjective recovery; hope, empowerment; confidence, connectedness with others, reliance	Internal consistency α =0.94 (intrapersonal subscale) and 0.77 (interpersonal subscale); Reliability Intra, r= 0.87; Inter, r= 0.77	Construct validity Intra, r= -0.83; inter, r= 0.52
12	WASI (*Wechsler, 1999*) 1 study: Wright et al., 2020	2 IQ tasks (verbal IQ and performance IQ) were used from the WASI	IQ (measure of neurocognition)	Not reported	Not reported
13	MAAS (*Brown & Ryan, 2003*) 1 study: Ubeda-Gomez et al., 2015	15 items Self-reported scale Response: Likert scale of 1-6	The dispositional capacity of awareness or attention to the experience of the present moment in daily life	Cronbach’s alpha = 0.89	Not reported
14	BDI-II (*Beck, Steer & Brown, 1996)* 2 studies: Perivoliotis et al., 2009; Farrelly et al., 2016	21 item- scale Self-report Response: 4-point Likert scale (0- symptom not present, to 3- present with significant distress)	Symptoms of depression	Not reported	Validated in patients with schizophrenia
15	BAI (*Beck & Steer, 1974*) 2 studies: Perivoliotis et al., 2009; Farrelly et al., 2016	21 item- scale Self-report Response: 4-point Likert scale (0- symptom not present, to 3- present with significant distress)	Symptoms of anxiety	Not reported	Validated in patients with schizophrenia
16	SCLF (*Strauss & Carpenter, 1974*) 1 study: Perivoliotis et al., 2009	9 item- questionnaire Interviewer-scored Not described	Levels of social and occupational functioning	Not reported	One of the most commonly used validated questionnaires of functioning
17	QoL Inventory (*Frisch, 1994*) 1 study: Perivoliotis et al., 2009	32-item Self-report Measures subjective functioning on 16 life domains	Satisfaction with life	Not reported	Not reported
18	BCIS (*Beck et al., 2004)* 1 study: Perivoliotis et al., 2009	15 items Self-report 2 dimensions of cognitive insight: Self-reflectiveness and self-certainty	Cognitive insight- the ability to question one’s beliefs, consider alternative explanations for one’s experiences and accept that beliefs are fallible.	Not reported	Validated
19	PDS (*Foa et al., 1997*) 1 study: Farrelly et al., 2016	49 items Total score ranging from 0 to 51 Response scale not described	A checklist of potentially traumatising events and an indication of the distress, intrusive thoughts, avoidance and hyperarousal in the last month	Not reported	Not reported
20	SCID-D (*Steinberg, 1994*) 1 study: Farrelly et al., 2016	Structured clinical interview for DSM-IV dissociative disorders 9 items	Depersonalisation symptoms	Not reported	Not reported
21	IPA (*Smith, Flowers, & Larkin, 2009*) 1 study: Bacon & Kenedy, 2014	Telephone interviews	Interpretative Phenomenological Analysis of semi-structured telephone interviews - detailed scrutiny of interview transcripts, development of conceptual themes, and repeating this with each set of interview data before superordinate and subordinate themes accommodating all experiences were produced.	Qualitative	Qualitative
22	HGSHS: A (*Shor & Orne, 1962*) 1 study: Humpston et al., 2016	Short hypnotic induction session followed by a series of 12 suggestions and a de-induction procedure 3 psychometric factors: ideomotor; challenge, cognitive	Compartmentalisation- type dissociative experiences through suggestion	Cronbach’s alpha 0.79	Well-validated

There is a huge variation in the degree of detail in which they were described by the authors of the articles included in this literature review, and also with regards to reporting aspects related to the reliability and validity of the instruments used.

## 6 Discussion

Studies that looked at the relationship between trauma and dissociation in people with psychosis demonstrate that patients with psychosis who have had traumatic experiences in childhood score higher on the Dissociative Experiences Scale (DES) than those who have not experienced traumas (26).

Classically but not always, dissociative experiences occur in response to psychological trauma. Watkins and Watkins referred to dissociation as an organising principle which allows people to adapt, thus moving the focus from a pathological perspective to a constructive potential and adaptive function of dissociation (41). Bowins expressed his view that dissociative manifestations can buffer disturbing emotional states, facilitating adaptive coping; they are employed by individuals to protect themselves against stress, therefore aiding recovery (42, 43). This shift in the way dissociation is now seen, and our clinical observations that people who are recovering from an episode of psychosis, are at the core of our initiative to carry out this overview of the existent literature looking at the usage of dissociation in the process of recovery from psychosis.

The review brings together a diversity of international research and theoretical views on the phenomenology of dissociation, psychosis and recovery. Due to the diverse designs of the studies and articles included, a narrative synthesis approach was more appropriate in order to try and understand the views on these three concepts and how they may be linked to each other. While none of them includes studies or views on the relationship between dissociation and recovery from psychosis, they do provide some perspectives and findings that could guide the discussion on this pioneering topic and inform and stimulate specific research on the matter.

The findings of this review (see **Table 6**) indicate that the prevalence of the derealisation syndrome is not different between the groups of participants diagnosed with schizophrenia and those without this diagnosis (18). An important finding showed that patients recovered from hallucinations had a significantly higher mean DES-II score than the nonclinical control group (t=11·130, p=0·009) and that the participants with psychotic disorder who had never had hallucinations, had a significantly higher score on the DES-II scale than the participants in the non-clinical control group (t=5·668, p=0·007) (19). Other findings were that compartmentalisation-type dissociation did not predict psychosis-like experiences and a *post hoc* cluster analysis indicated that detachment-type dissociation and absorption are largely distinct from psychosis-like experiences and do not reflect similar constructs (6).

**Table 6:** Results and limitations.

Nr	Author/Year	Results	Limitations
1	Longden et al. (2020)	• Dissociative phenomena are significantly related to positive symptoms and disorganisation. • Associations with negative symptoms were of smaller magnitude or nonsignificant. • The effects considered in the review were observed across both clinical and nonclinical samples.	• Impact on the magnitude of effects due to patients being likely more symptomatic than nonclinical participants; • Fundamental differences in the constructs assessed by different measures; • The search strategy was limited to peer-reviewed English-language studies; • The same studies examined multiple psychotic experiences within the same sample
2	Farrelly et al. (2016)	No results available as this paper presents the protocol for a study to assess the feasibility and acceptability of a brief cognitive behavioural therapy intervention for individuals who have depersonalisation symptoms in the context of psychotic symptoms.	
3	Hwu et al. (1981)	• The prevalence of the derealisation syndrome at initial evaluation is not different between the clinical (patients with schizophrenia) and the non-clinical groups.	not reported
4	Perona-Garcelan et al. (2008)	• The attention of subjects with hallucinations was more self-focussed than the nonclinical group; • A positive correlation (p<0.05) between self-focusing and dissociative experiences in subjects with hallucinations; • Depersonalisation - the only factor predicting auditory hallucinations (F[1,66] = 113.366, p =0.000); • Patients recovered from hallucinations had a significantly higher mean DES-II score than the nonclinical control group (T = 11.130, p = 0.009); • The subjects with psychotic disorder who had never had hallucinations, had a significantly higher score on the DES-II scale than the subjects in the nonclinical control group (T = 5.668, p =0.007). • Absorption - the patients recovered from hallucinations and those who had never had hallucinations had significantly higher scores than the nonclinical control group (T = 18.465, p = 0.012; T = 8.586, p = 0.007, respectively);	• Small sample size; • Limited in generalizability; • Only used single dissociative instruments.
5	Perona-Garcelan et al. (2012)	• Significant differences between the groups (p <.001) regarding depersonalisation, absorption and metacognitive variables • Both absorption and depersonalisation were positively associated with all Metacognition Questionnaire -30 subscales, and also with the total score (p <.01). • The variable with the most predictive power for hallucinations (scores on the PANSS) of all those used in this study was depersonalisation [F (1, 122) = 101.472, p <.001].	• Unable to establish any causal relationships between the variables studied; • Uncontrolled variable (schizophrenic patients were on antipsychotic medication while the rest of the subjects were not) that could affect the dependent variables; • Under reported symptoms; • Potential bias by the relationship between anxiety and depression and the dissociative variables; • Difficulties in understanding some of the items.
6	Wright et al. (2020)	• Metacognitive ability was a significant predictor of functional capacity, R2 = 0.23, F(1, 131)=38.98, p<.001; and functional outcome, R2 = 0.104, F(1, 133)=15.39, p<.001; and subjective recovery outcome in FEP R2 = 0.39, F(3, 57)=11.55, p<.001. • Metacognitive control was a significant predictor of functional capacity, R2 = 0.11, F(1, 130)=16.16, p<.001. • Metacognitive experience was a significant predictor of functional capacity, R2 = 0.101, F(1, 131)=14.6, p<.001; and functional outcome, R2 = 0.03, F(1, 132)=4.15, p=.04. • The FEP group demonstrated more accurate metacognitive experience (appraisal of experience), and higher scores on metacognitive monitoring compared to controls.	• The authors combined FEP and healthy control group in order to increase sample size and range of scores; • Individuals who typically engage in research studies tend to be higher-functioning, caution should be taken when applying these results to a lower functioning group.
7	Rosen et al. (2017)	• A highly positive correlation (p<0.001) between absorption and hallucinations, thought disorder; • 2 subtypes of absorption within the sample: Cluster One- Attenuated Ego Boundaries (AEB) - 55 participants both clinical and non-clinical (48%); Cluster Two - Stable Ego Boundaries (SEB) - 60 participants (52%). • A significant increase in PANSS positive, cognitive, excitement, depression factor scores in the AEB cluster compared to the SEB cluster; no significant differences between cluster groups in PANSS negative factor scores.	• Small sample size; • Results based largely on self report - limited generalizablity
8	Úbeda-Gómez et al. (2015)	• Distress caused by the voices correlated positively with self-focussed attention (private and public) (p< 0.001) and negatively with mindfulness (p<0.001); • A negative correlation was also found between mindfulness and self-focussed attention - private (p<0.05) and public (p<001); • Public self-focus was the only factor predicting distress caused by the voices [R2 = 0.25, F(1, 50)=17.66, p<.001;	•No causal relationships because of being a correlational study; •Not clearly isolated differences among groups with regard to the variables involved.
9	Humpston et al. (2016)	• Detachment and absorption predicted levels of delusional ideation and anomalous perceptual experiences; • Compartmentalisation did not predict psychosis-like experience; • Detachment and absorption are largely distinct from psychosis-like experience and do not reflect similar constructs.	•Not possible to determine the direction of causality; •The results may not always fully translate to other cultural settings.
10	Lynch et al. (2018)	Participants reported that through art therapy, they were able to build up relationships, connect with others, sustain participation and therapeutic engagement and experience therapeutic change.	•The service users were not involved in the design or execution of the study; •Not all participants brought artwork to discuss during the interview.
11	Bacon & Kenedy (2014)	• A model of the Theory of Structural Dissociation of the Personality (TSDP) suggests that the difference between psychosis and dissociation is circumstantial, dependent on the structural dissociation and mental level of an individual’s personality (or parts thereof) at that time. It validates suggestions of a continuum-based approach to psychosis and dissociation as traumatic reactions.	•Difficult to disentangle the authors own beliefs from the experiences and understandings of the participants; •Factors such as participant nationality, profession and recruitment organisation may have influenced the findings.
12	Ross & Keyes (2009)	The authors predicted that the dissociative subtype of schizophrenia affects in the range of 25–40% of individuals meeting DSMIV-TR diagnostic criteria for schizophrenia. They diagnosed dissociative schizophrenia in 22% of the 50 cases interviewed. The percentage of cases assigned to the proposed dissociative subtype was within the range of the research-based prediction.	•The interviews were conducted by foreigners using translators; •Vague histories given by the participants; •Potential memory problems due to institutionalisation, ECT and medications; •Limited size of sample; •The lack of knowledge of dissociation making questions unclear to the participants.
13	Lysaker et al. (2020)	• No statistics reported; • Deficits in metacognition commonly occur in schizophrenia and are related to basic neurobiological indices of brain functioning; • The capacity for metacognition in schizophrenia is positively related to a broad range of aspects of psychological and social functioning when measured concurrently and prospectively; • Metacognitive Reflection and Insight Therapy (MERIT) has the potential to treat fragmentation and promote recovery.	•Difficulty measuring the extent of metacognitive deficit; •Lack of long term, longitudinal studies.
14	Lysaker et al. (2018)	•Review of major theories of alterations in self- experience in schizophrenia; • Results: The authors argue that research on metacognition suggests that reduction in metacognitive function may partially explain the occurrence of these difficulties and also explain how their resolution contributes to recovery.	
15	Kumar et al. (2018)	•50% reduction of PSYRATS score on the items related to the beliefs about origins of voices, intensity of distress, interference with life and controllability; •May be effective in patients who are not receiving antipsychotic treatment.	•Limited generalizability of the findings; •No discussion about the impact of the disappearance of the psychotic experiences; •No standard measure to assess the subjective rating of patient about recovery; •No tool for assessment of patient’s metacognitive capacities.
16	Perivolioitis et al. (2009)	• Psychological intervention can be adapted to successfully treat patients with schizophrenia who lack insight; • The cognitive formulation of negative symptoms provides a useful roadmap; • The importance of experiential learning in driving both behaviour change and belief modification.	•Under reporting symptoms and guarded
17	Pec et al. (2020)	The metacognitive approach might provide operational definitions for psychoanalytic concepts in schizophrenia related to self-disturbance and the emotional, cognitive, and social disruptions associated with psychoanalytic understanding of fragmentation.	
18	Bob & Mashour (2011)	• Significant overlaps between the symptomatology and experimental data regarding dissociative processes and schizophrenia. • Although direct evidence is lacking, the investigation of dissociative processes may be beneficial for understanding certain types of schizophrenia.	
19	Ross (2006)	It proposes a dissociative subtype of schizophrenia.	

These findings are consistent with the views that dissociation phenomena can spread on a continuum of distress and disability (7), ranging from nonpathological experiences to chronic and extremely disabling conditions (44).

## 7 Limitations

**Table 6** summarises the limitations of the studies as reported by the authors.

One of the major limitations is the small number of publications included in the review and their very diverse nature. Because research in this field is difficult to undertake due to the difficulty to conceptualise dissociation and the overlapping of the phenomenological manifestations of dissociation and psychosis (35), we identified a very small number of publications that could be included in our review, despite the initial identification of a large number of publications in the world literature. None of them put together, in the same conceptualisation or hypothesis, dissociation and the recovery from an episode of psychosis, therefore this matter remains unstudied at this time. For the purpose of this review of the literature, we did not differentiate between the psychotic conditions because they are a large group of nosological entities. Although characterised by similar types of symptoms, psychotic conditions can differ in their manifestations, severity, response to treatment, prognosis and duration (45). This is an aspect that may be helpful to consider in a future research project.

We acknowledge the fact that our review of the literature only focussed on the relationship between dissociation and recovery from psychosis, and did not evaluate the factors that can influence the complex interaction between dissociation and psychosis, such as treatment, psychotherapy and history of trauma. The role of psychotherapeutic interventions in the recovery from psychosis is essential (46–48) but this was not evaluated during our survey. Another important factor is the treatment and response to treatment. There are instances where recovery can be difficult to achieve due to resistance to treatment. Resistance to drug therapy is reported in approximately 30–50% of patients with schizophrenia (49, 50). Panov (2022) (51) conducted a study that looked at the relationship between the degree of dissociation and resistance to therapy. The findings showed a high degree of dissociation in patients with resistant schizophrenia compared with those in remission. It has been demonstrated that those with a high degree of dissociation have a more severe course of illness (52). Consideration needs to be given also to the mechanism of action of antipsychotics as pharmacotherapy is crucial in the process of recovery from psychosis. An interesting hypothesis about the mechanism of action of atypical antipsychotics is proposed by Kapur and Seeman (2001) (53) explaining the “atypical” antipsychotic effect of the second generation antipsychotics. According to that, the fast dissociation from the D2 receptor makes an antipsychotic more accommodating of physiological dopamine transmission, permitting an antipsychotic effect without major side effects. This will improve compliance with treatment and subsequently facilitate the alleviation of symptoms and the process of recovery (54). Trauma is another factor that mediates the relationship between dissociation and psychosis (55, 56) and would need to be addressed in order to facilitate recovery. An interesting finding was reported by Van der Linde et al. (2023) who conducted a study that investigated the role of dissociation related beliefs about memory in trauma-focussed treatment. The results showed that dissociation-related beliefs do not influence the outcome of trauma-focussed treatment (57), The authors of some of the included studies report limitations to their projects. These, we believe, are important sources of learning and they could inform further research and stimulate curiosity to explore whether dissociative mechanisms are used by people with psychosis when they recover from an episode of illness.

One of the main learning points is that there are fundamental differences in the conceptualisation of the notions explored and their assessment by different measures (7). There is no consistency in reporting dissociation scale scores in the papers included in this review and therefore a cross-sectional comparison of the outcomes was not possible. Although the articles included report studies conducted in different countries, the search strategy was limited to those published in English. Although the population samples used for these studies include a wide range of ages and sources, the potential influence exercised by the cultural factor onto the dissociative experiences suggests that the results communicated by the Western studies may not always fully translate to other cultural settings (6). The small sample size studies (19, 22) and the cross-sectional design (6, 19–24) limited the generalizability of findings and the possibility to extract causal relationships among the variables studied. Another factor that could have influenced the findings, could be the recruitment methodology: approached by clinicians based on diagnoses (17, 19–21, 23, 25, 26), advertisement within the local community (6, 21), random selection from an urban university medical centre and neighbouring communities (22); from research registers (17). Many factors such as participant nationality, profession, recruitment organisation may have influenced the findings and for this reason they cannot be considered representative (5).

## 8 Conclusion

Dissociation is a complex phenomenon involving different mechanisms that can modulate both the psychopathological processes underlying psychosis and recovery. The idea of integrative dissociation, suggested by Bacon and Kennedy (5), actually opens up the way to a new conceptualisation of dissociation, with potential impact on therapeutic intervention.

The process of reviewing the existing scientific literature in the field of dissociation and recovery from psychosis has been useful for charting the direction that future research projects will take. The putative association which we have raised, between dissociation and recovery from psychosis, has not previously been researched.

The literature is extremely diverse and dissociation is a phenomenon with many facets, difficult to measure unitarily, but which can be conceptualised very specifically through its processes. As a future project, a review of the different conceptual- definitions of dissociation, would seem of value given the diversity of its conceptualisation in the material presented.

Further research is needed to observe what happens to dissociative phenomena throughout the evolution of psychosis and not just in the acute phase of this illness. This work could helpfully include qualitative, patient experience studies and outcome research using tools identified in this review.

This overview of the literature should be considered a preliminary attempt to explore dissociation in recovery as we believe it to be a topic of clinical interest in a time of change in how therapeutic interventions are provided within the mental health services. It would be of interest to replicate the survey and evaluate also the factors which we did not include in this review.

## Data availability statement

The original contributions presented in the study are included in the article/supplementary material. Further inquiries can be directed to the corresponding author.

## Author contributions

CC: Writing – review & editing, Writing – original draft, Validation, Supervision, Project administration, Methodology, Formal analysis, Data curation, Conceptualization. RM: Writing – review & editing, Validation, Conceptualization. SC: Writing – review & editing, Investigation, Formal analysis. MZ: Writing – review & editing, Investigation, Formal analysis. RK: Writing – review & editing, Investigation, Formal analysis. KR: Writing – review & editing, Validation. CS: Writing – review & editing, Validation, Supervision. JW: Resources, Writing – review & editing.
